# Value of [^18^F]AlF-NOTA-FAPI PET/CT in guiding radiotherapy planning for stage I-IIIC lung cancer: A comparison with contrast-enhanced CT and [^18^F]FDG PET/CT

**DOI:** 10.1007/s00259-025-07753-7

**Published:** 2026-02-05

**Authors:** Jingjie Qin, Chengqiang Li, Yong Huang, Yuqin Jin, Xiaoshan Liu, Xudong Hu, Jian Zhu, Junya San, Hongbo Wu, Xue Meng, Jinming Yu, Yuchun Wei

**Affiliations:** 1 Department of Radiation Oncology, Shandong Cancer Hospital and Institute, Shandong First Medical University and Shandong Academy of Medical Sciences, Jinan, Shandong China; 2 Department of Radiation Oncology Physics & Technology, Shandong Cancer Hospital and Institute, Shandong First Medical University and Shandong Academy of Medical Sciences, Jinan, Shandong China; 3 Shandong Provincial Key Medical and Health Laboratory of Pediatric Cancer Precision Radiotherapy, (Shandong Cancer Hospital), Jinan, Shandong China; 4 Department of Radiology, Shandong Cancer Hospital and Institute, Shandong First Medical University and Shandong Academy of Medical Sciences, Jinan, Shandong China; 5 Department of Radiology, Shandong First Medical University and Shandong Academy of Medical Sciences, Jinan, Shandong China; 6 Department of Nuclear Medicine PET Center, Cancer Hospital and Institute, Shandong First Medical University and Shandong Academy of Medical Sciences, Jinan, Shandong China; 7 Department of Radiation Oncology, Shandong Provincial Key Laboratory of Radiation Oncology, Shandong Cancer Hospital and Institute, Shandong First Medical University and Shandong Academy of Medical Sciences, Jinan, Shandong China; 8 Shandong Cancer Hospital and Institute, Shandong First Medical University and Shandong Academy of Medical Sciences, No. 440 Jiyan Road, Jinan, Shandong 250117 China

**Keywords:** FAPI PET/CT, Lung cancer, Radiotherapy, Gross tumor volume, IMRT/IMPT

## Abstract

**Purpose:**

To evaluate the usefulness of [^18^F]AlF-labeled fibroblast activation protein inhibitor (FAPI), chelated with NOTA (denoted as [^18^F]AlF-NOTA-FAPI), positron emission tomography/computed tomography (PET/CT) for radiotherapy planning in lung cancer, we compared it with contrast-enhanced CT (CE-CT) and [^18^F]FDG PET/CT.

**Materials and methods:**

In this secondary analysis of a prospective trial, patients with stage I-III lung cancer who underwent [^18^F]AlF-NOTA-FAPI and CE-CT or [^18^F]FDG PET/CT scans within 2 weeks were selected. Gross tumor volume (GTV), clinical tumor volume (CTV), and planning tumor volume (PTV) were drawn for the primary tumor (GTVp) and involved lymph nodes (GTVnd) based on CE-CT, [^18^F]AlF-NOTA-FAPI PET/CT and [^18^F]FDG PET/CT. Organs at risk were evaluated and compared in intensity-modulated radiation therapy (IMRT) and intensity-modulated proton therapy (IMPT) plans.

**Results:**

Fifty-one patients (median age, 64 years; 38 males [74.5%]) were evaluated. Statistically significant differences in GTVs based on CE-CT, [^18^F]AlF-NOTA-FAPI PET/CT, and [^18^F]FDG PET/CT were found between each pair (all *P* < 0.05), except that no difference in GTVp was found between [^18^F]AlF-NOTA-FAPI and [^18^F]FDG PET/CT (*P =* 0.558). CE-CT-based GTVp values were significantly larger than those based on [^18^F]FDG PET/CT and [^18^F]AlF-NOTA-FAPI PET/CT due to the presence of obstructive pneumonia (*n* = 18; all *P* < 0.05). PTVall-IMRT based on CE-CT (351.98 ± 26.87) was significantly larger than that based on [^18^F]AlF-NOTA-FAPI PET/CT (329.98 ± 26.21; *P* = 0.03). PTVall-IMPT based on CE-CT (212.74 ± 18.73) was significantly larger than those based on [^18^F]FDG PET/CT (204.26 ± 19.34) and [^18^F]AlF-NOTA-FAPI PET/CT (196.99 ± 18.38), with *P*-values of 0.046 and 0.011, respectively. For IMRT, [^18^F]AlF-NOTA-FAPI PET/CT-based plans significantly reduced the radiation doses received by the heart, contralateral lung, and both lungs (all *P* < 0.05).

**Conclusion:**

[^18^F]AlF-NOTA-FAPI PET/CT shows promise as a valuable tool for radiotherapy planning in lung cancer, providing accurate target delineation and improved sparing of critical organs.

**Trial registration:**

This study was approved by the Clinical Research Ethics Committee of our institution.

**Supplementary Information:**

The online version contains supplementary material available at 10.1007/s00259-025-07753-7.

## Introduction

Radiotherapy is a critical modality in the treatment of lung cancer. Evidence-based indications for radiotherapy apply to 77% of all lung cancer patients [1]. However, an evidence-based population modelling study of the overall lung cancer population estimated that radiotherapy improves 5-year loco-regional control and overall survival by only about 8.3% and 4.0%, respectively [2], indicating that substantial room for optimization remains. One important reason for this limited benefit is suboptimal target delineation, which can cause both geographic miss and unnecessarily large target volumes, thereby increasing normal-tissue toxicity and compromising the therapeutic ratio [3]. In addition, larger irradiation volumes and greater low-dose exposure of the lung and circulating blood increase the risk of radiation-induced lymphopenia, which is strongly associated with poorer outcomes, particularly in patients receiving concurrent or sequential chemotherapy and immunotherapy [4, 5]. These considerations have driven wider use of highly conformal techniques and stereotactic body radiotherapy (SBRT), even for selected larger tumors and non-typical histologies such as small-cell lung cancer (SCLC) and other neuroendocrine tumors. However, for the many patients who still receive conventionally fractionated thoracic radiotherapy, accurate imaging-guided target delineation remains a major unmet clinical need.

Imaging plays a pivotal role in the precision of radiotherapy delivery. High-resolution contrast-enhanced CT (CE-CT) effectively visualizes tumor structure, shape, and density, and is primarily used to guide radiotherapy based on CT density changes. However, CE-CT sometimes struggles to differentiate tumors from benign conditions such as granulomatous inflammation [6], tuberculosis [7] or obstructive pneumonia [8], and often misses small metastatic lymph nodes. Central lung cancer is frequently accompanied by obstructive pneumonia, yet CE-CT cannot accurately distinguish the boundary between the tumor and atelectasis [9], resulting in imprecise radiotherapy target delineation and severe radiation-induced damage due to excessively large target volumes. [^18^F]FDG PET/CT can differentiate between obstructive pneumonia and tumor boundaries, aiding in target delineation [10]. However, [^18^F]FDG uptake reflects glucose metabolism and is difficult to distinguish inflammation from malignancy. Conversely, [^18^F]FDG PET/CT may show low or absent uptake in certain lung cancer subtypes, such as lepidic adenocarcinoma, typical and atypical carcinoid tumors and mucinous adenocarcinoma, thereby underestimating or even omitting tumor extent despite the presence of viable tumor [11–13]. Taken together, these limitations highlight the need for complementary imaging approaches that can more clearly define the gross tumor volume (GTV), include small and metabolically indolent lesions, and exclude non-malignant disease.

Fibroblast activation protein inhibitor (FAPI) PET imaging agents target fibroblast activation protein (FAP), a key component of the tumor microenvironment [14, 15], enabling imaging of various solid tumors with excellent tumor-to-background contrast [16–18]. Studies have confirmed that FAP as an antigen target can be visualized in vivo using ^68^Ga/^18^F-FAPI PET/CT, improving tumor detection and diagnosis rates [6, 17] aiding in the differentiation of lung cancer from pneumonia [8] and facilitating radiotherapy target delineation [19]. However, the utility of [^18^F]AlF-NOTA-FAPI PET/CT to guide radiotherapy target delineation in lung cancer remains unclear, particularly when compared directly with CE-CT and [^18^F]FDG PET/CT across different radiotherapy techniques.

We hypothesize that radiotherapy targets derived from [^18^F]AlF-NOTA-FAPI PET/CT will be more optimal for lung cancer. In this study, we explored the clinical applications of [^18^F]AlF-NOTA-FAPI PET/CT in terms of radiotherapy target accuracy, target volume, and dose to normal tissues and organs, and compared these with CE-CT and [^18^F]FDG PET/CT.

## Materials and methods

### Participants

This planning and dosimetric study represents a secondary analysis of a single-centre prospective imaging trial entitled “[^18^F]AlF-NOTA-FAPI PET/CT imaging in patients with tumors”. The protocol was approved by the institutional review board of the affiliated Cancer Hospital of Shandong First Medical University (SDZLEC2021-112-02). The primary diagnostic accuracy results of the parent trial have been reported previously [6]. Participants were consecutively recruited from December 2020 to April 2022, and all patients provided written informed consent before inclusion in the study.

**Fig. 1:**
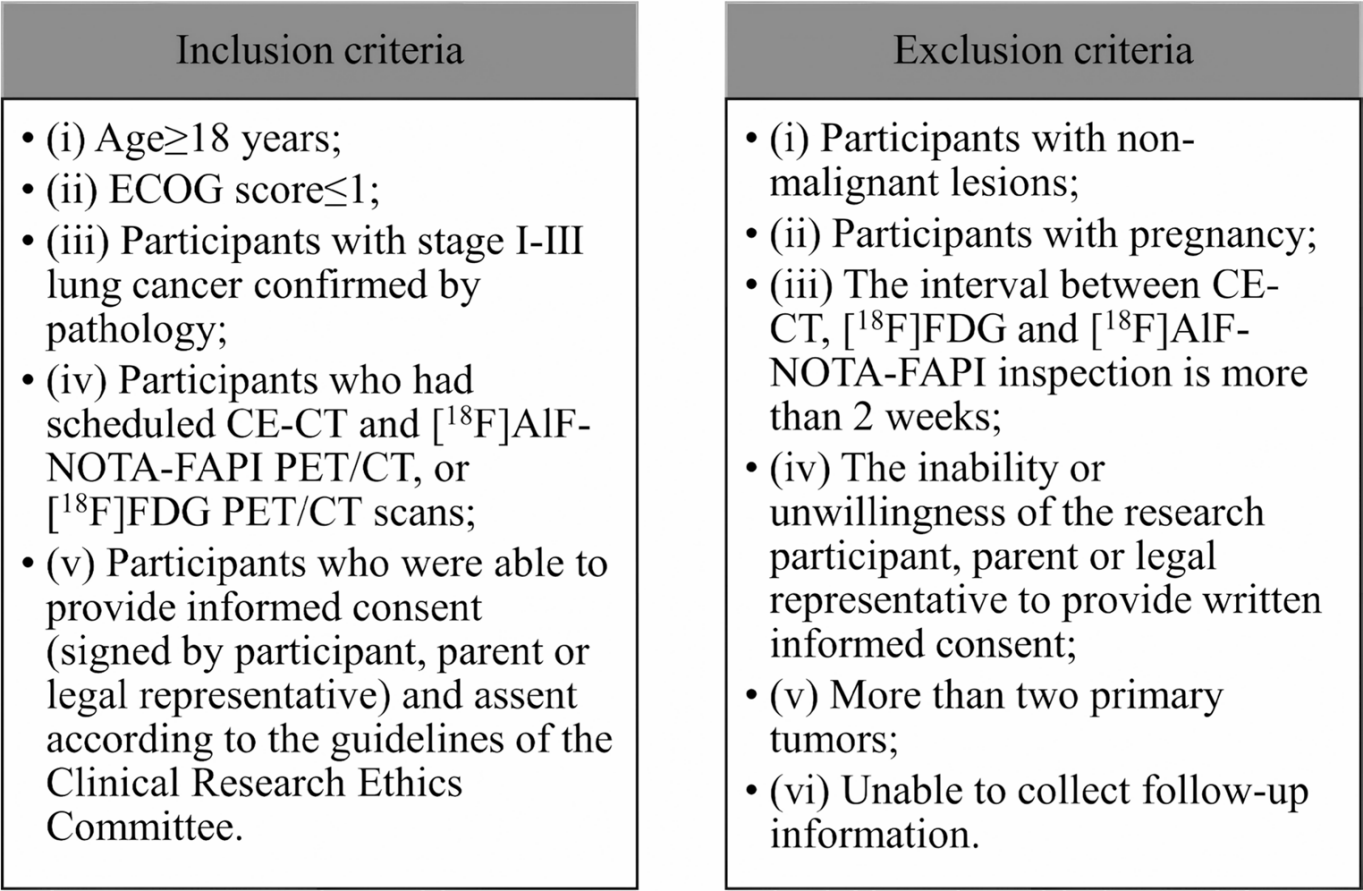
Inclusion and exclusion criteria. ^18^F=Fluorine 18, FAPI = Fibroblast activation protein inhibitor, FDG = fluorodeoxyglucose, CE-CT = Contrast-enhanced CT

The inclusion and exclusion criteria are presented in Fig. 1.

### Preparation of [^18^F]AlF-NOTA-FAPI and [^18^F]FDG

1,4,7-Triazacyclononane-N, N’,N”-triacetic acid (NOTA)-FAPI-04 (Nanchang Tanzhen Biotechnology Co., Ltd.) was radiolabeled with Al^18^F, and [^18^F]AlF-NOTA-FAPI and [^18^F]FDG were synthesized according to reported processes [20].

### CT and PET/CT imaging

All CE-CT examination were performed using four helical CT scanners. The detailed parameters of scanning and reconstruction are consistent with what was previously reported [21].

[^18^F]AlF-NOTA-FAPI and [^18^F]FDG PET/CT scans were obtained at intervals of more than 20 h and within 14 days of each other. Before [^18^F]FDG PET/CT scanning, participants were instructed to fast for at least 6 h, and a normal blood glucose level in peripheral blood was ensured. Fasting blood glucose measurement was not required or requested before [^18^F]AlF-NOTA-FAPI PET/CT imaging. Participants received an intravenous injection of 4.81 MBq/kg (0.12 mCi/kg) [^18^F]FDG/[^18^F]AlF-NOTA-FAPI and rested for about 60 min before imaging. Scanning was then performed with an integrated in-line PET/CT system (GEMINI TF Big Bore; Philips Healthcare). Whole-body CT scans were acquired for attenuation correction using a low-dose protocol (300 mAs, 120 kV, a 512 × 512 matrix, rotation time of 1.0 s, and pitch index of 0.688; reconstructed with a soft-tissue kernel to a section thickness of 5 mm, CTDI of 8.7mGy, DLP of 901.3mGy*cm). Subsequently, PET data were acquired in 3-dimensional mode with a 200 × 200 matrix with 1-min imaging time per bed position. After randoms, decay and scatter correction, the data were reconstructed (Body-ctac-SB. Lstcln, Biograph 3D iterative reconstruction software [version, Release 3.6.2], TOF correction). The obtained images were attenuation-corrected with the transmission data from CT. The attenuation-corrected PET images, CT images, and fused PET/CT images, displayed as coronal, sagittal, and transaxial slices, were viewed and analyzed using a Nuclear Medical Information System (Beijing Mozi Healthcare, Ltd.).

### Target volume delineation

The target areas of non-small cell lung cancer (NSCLC) [22] and SCLC [23] were delineated respectively according to the ESTRO ACROP guidelines. Gross tumor volumes of the primary tumor (GTVp) and involved lymph nodes (GTVnd) were contoured separately. Clinical target volumes (CTVp and CTVnd) were generated by expanding GTVp and GTVnd, respectively. For squamous cell carcinoma, an isotropic margin of 6 mm was applied; for adenocarcinoma, 8–10 mm; and for SCLC, 5–6 mm. CTVp and CTVnd were then each expanded by 5 mm to form the planning target volumes of the primary tumor (PTVp) and lymph nodes (PTVnd). For treatment planning and plan evaluation, PTVp and PTVnd were combined to form a composite PTV (PTVall), with overlapping regions counted only once.

For all patients, GTVp and GTVnd were manually delineated on contrast-enhanced large-bore simulation CT scans by an experienced thoracic radiation oncologist. Co-registered CE-CT, [^18^F]AlF-NOTA-FAPI PET/CT and [^18^F]FDG PET/CT images were reviewed side by side and used as complementary information to refine tumor boundaries and identify metastatic lymph nodes. No fully or semi-automatic contouring methods were used, and no fixed SUV-threshold–based segmentation was applied on PET/CT. Instead, PET uptake patterns were interpreted visually in the anatomical context of the CT images to guide manual contour adjustment. All contours were subsequently reviewed and, where necessary, modified in consensus with a second senior radiation oncologist, so that the final target volumes reflected a two-expert consensus.

### Radiotherapy planning

The treatment plans were calculated using the Eclipse™ treatment planning system (version 16.1, Varian Medical System, Palo Alto, CA). The dose calculation grid size was 2.5 mm. Photon IMRT and proton IMPT plans were generated for each patient on a Varian TrueBeam linear accelerator and a ProBeam proton therapy system, respectively. IMRT plans were prescribed 60 Gy in 30 fractions and normalized such that at least 95% of the PTV received the prescription dose. IMPT plans employed pencil-beam scanning with multi-field optimization and were planned so that 95% of the CTV received 60 Gy in the nominal scenario; robust optimization with 5-mm setup and 3.5% range uncertainties ensured CTV coverage ≥ 90% in the second-worst evaluated scenario. For each modality, CE-CT-, [^18^F]FDG PET/CT- and [^18^F]AlF-NOTA-FAPI PET/CT-based plans were created using identical beam arrangements and the same overall planning strategy, with only minor, case-specific adjustments of optimization objective weights to meet the predefined target coverage and organ-at-risk constraints. All IMRT and IMPT plans were generated by a single experienced medical physicist and reviewed by the same thoracic radiation oncologist.

### Intensity modulated proton therapy (IMPT)

All treatment IMPT plans were robustly optimized with the goal of 95% of CTV receiving the prescription dose while minimizing dose to the organ at risks (OARs). Relative biological effectiveness, the ratio of absorbed doses between two modalities to have the same biological effect, is generally considered to be 1.1 for protons compared to photons. Robust optimization (3.5% calibration curve error and 5 mm setup uncertainty) was used to account for uncertainties and the interplay effect. All patients were treated with multi-field optimized (MFO) pencilbeam scanning IMPT on a Varian Probeam proton therapy system (Varian Medical Systems, Inc., Palo Alto, CA, USA). For the MFO approach, the nonlinear uniform proton optimizer (NUPO) was used. The energy range of the machine was from 70 to 244 MeV. Usually, 2 to 3 proton beams were used.

### Intensity modulated radiotherapy (IMRT)

Photon optimizer model in the Eclipse™ TPS was used for IMRT optimization, and analytical anisotropic algorithm (AAA) model was used for dose calculation. Approximately 5–7 coplanar 6MV photon beams were used for all IMRT plans implemented for a TrueBeam linear accelerator with a maximum dose rate of 600 MU/min. All treatment IMRT plans were robustly optimized with the goal of 95% of PTV receiving the prescription dose while minimizing dose to the OARs.

### Quality assurance for planning volume

An in-house developed dose-volume histograms (DVH) tool implemneted in Matlab version R2016a (The MathWorks Inc., Natick, MA, United States) software was used for comparing dose values of target and OARs for all plans. For OARs, the following parameters were evaluated: mean dose, V5, V10, V20 and V30 to both lungs, heart, ipsilateral lung and contralateral lung. For dosimetric parameters, target conformity index, homogeneity index and gradient index were calculated.

### Dice similarity coefficient (DSC)

To quantify spatial agreement between contours generated from different imaging modalities, the DSC was computed as $DSC=\frac{2\left|A\cap B\right|}{\left|A\left|+\right|B\right|}$, where A and B represent two contour volumes. DSC was computed between GTV contours (GTVp and GTVnd) derived from CE-CT, [^18^F]FDG PET/CT, and [^18^F]AlF-NOTA-FAPI PET/CT, yielding in three pairwise comparisons: CE-CT vs. FDG, CE-CT vs. FAPI, and FDG vs. FAPI.

### Statistical analysis

Descriptive statistical methods were used to compare the age and target volume values of CE-CT, [^18^F]FDG and [^18^F]AlF-NOTA-FAPI among participants. Normally distributed data were expressed as the mean ± SD, and non-normally distributed data were expressed as the median (IQR, 25%−75%). Comparisons of target volume and DVH parameters between the two groups were performed using a paired two-sample t test, and comparisons of non-normally distributed data between the two groups were performed using the McNemar test. The McNemar chi-square test with the four-grid table was used to compare the GTV consistency of CE-CT, [^18^F]FDG PET/CT and [^18^F]AlF-NOTA-FAPI PET/CT. One-way ANOVA was applied to evaluate DSC differences across contour-pair categories, followed by LSD post-hoc tests for pairwise comparisons when equal variance was confirmed by Levene’s test. Two-tailed *P* values < 0.05 indicated statistically significant differences. All analyses were performed using IBM SPSS 20.0 (IBM).

## Results

### Basic and clinical characteristics of participants with lung cancer

**Fig. 2:**
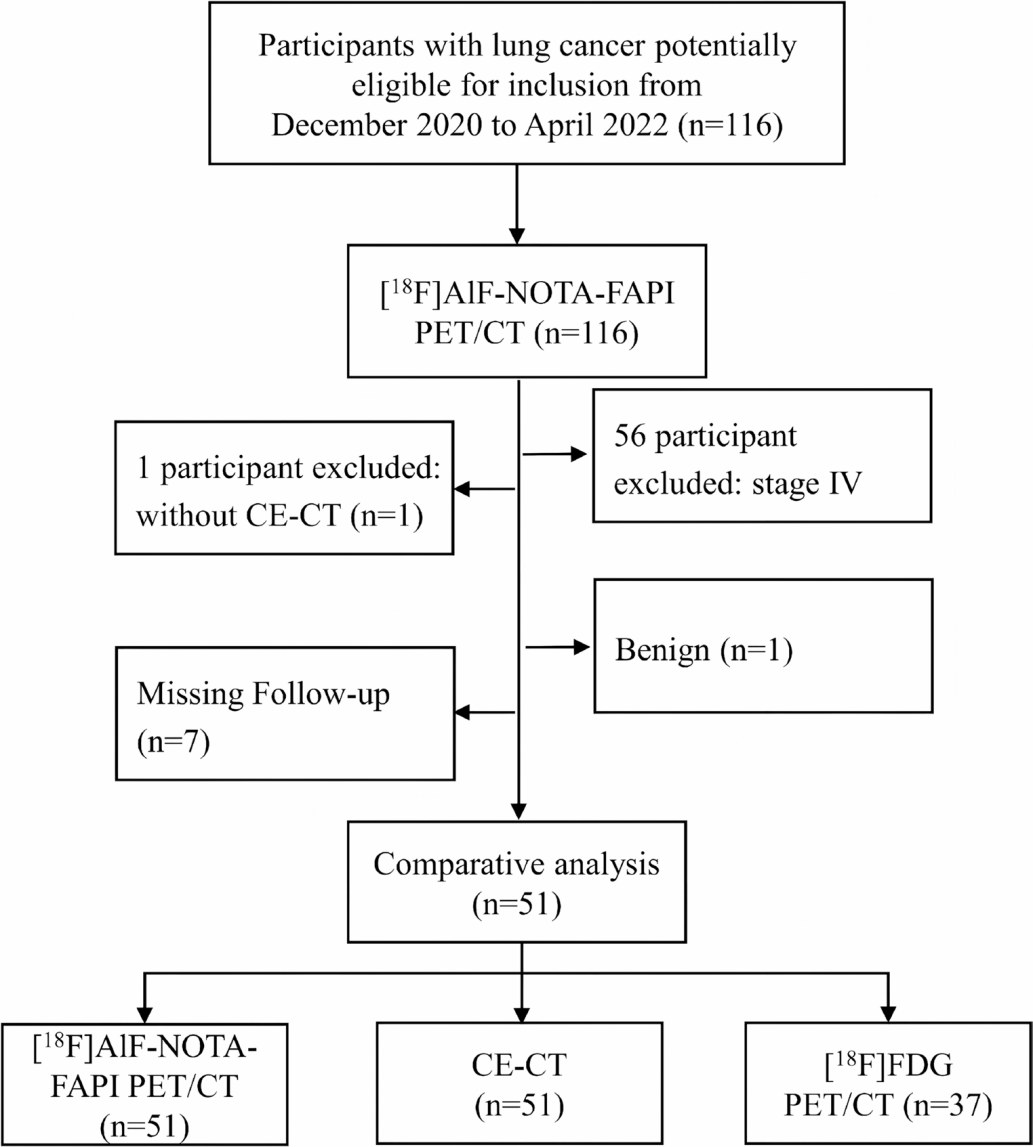
Study flow chart. ^18^F=Fluorine 18, FAPI = Fibroblast activation protein inhibitor, FDG = fluorodeoxyglucose, CE-CT = Contrast-enhanced CT

The final study sample included 51 participants (median age, 64 years [IQR, 56–69 years]; 38 [74.51%] male and 13 [25.49%] female) (Fig. 2). The basic and clinical characteristics of these participants are presented in Table 1.

**Table 1:** Participant characteristics

Characteristics	Value (*n* = 51)
Median age (y)	64 (range 43 ~ 75)
Sex	
M	38 (74.51)
F	13 (25.49)
ECOG performance status	
0	19 (37.26)
1	32 (62.74)
Pathologic subtype	
ADC	20 (39.21)
SCC	22 (43.14)
SCLC	7 (13.73)
Others	2 (3.92)
Obstructive pneumonia	
Yes	18 (35.29)
No	33 (64.71)
Stage, n (%)	
I	5 (9.80)
II	5 (9.80)
IIIA	27 (52.94)
IIIB	10 (19.61)
IIIC	4 (7.84)
Imaging, n (%)	
CE-CT	51 (100)
FAPI PET/CT	51 (100)
FAPI & FDG PET/CT	37 (71.55)
Method of confirmation	
Histopathologic results	20(39.22)
Follow-up results	31(60.78)
Systemic therapy modality, n (%)	
CCRT	11(21.57)
sCRT	15(29.41)
Others	25(49.02)
Immunotherapy, n (%)	
Yes	28(54.90)
No	23(45.10)

*M* male, *F* female, *ADC* adenocarcinoma, *SCC* squamous cell carcinoma, *SCLC* small cell lung cancer, Others including 2 cases of large cell lung cancer, *CE-CT* Contrast-enhanced CT, *FDG* fluorodeoxyglucose, *FAPI* fibroblast activation protein inhibitor, *CCRT* concurrent chemoradiotherapy, *sCRT* sequential chemotherapy and radiotherapy

### GTV consistency based on CE-CT, [^18^F]AlF-NOTA-FAPI PET/CT, and [^18^F]FDG PET/CT

**Fig. 3:**
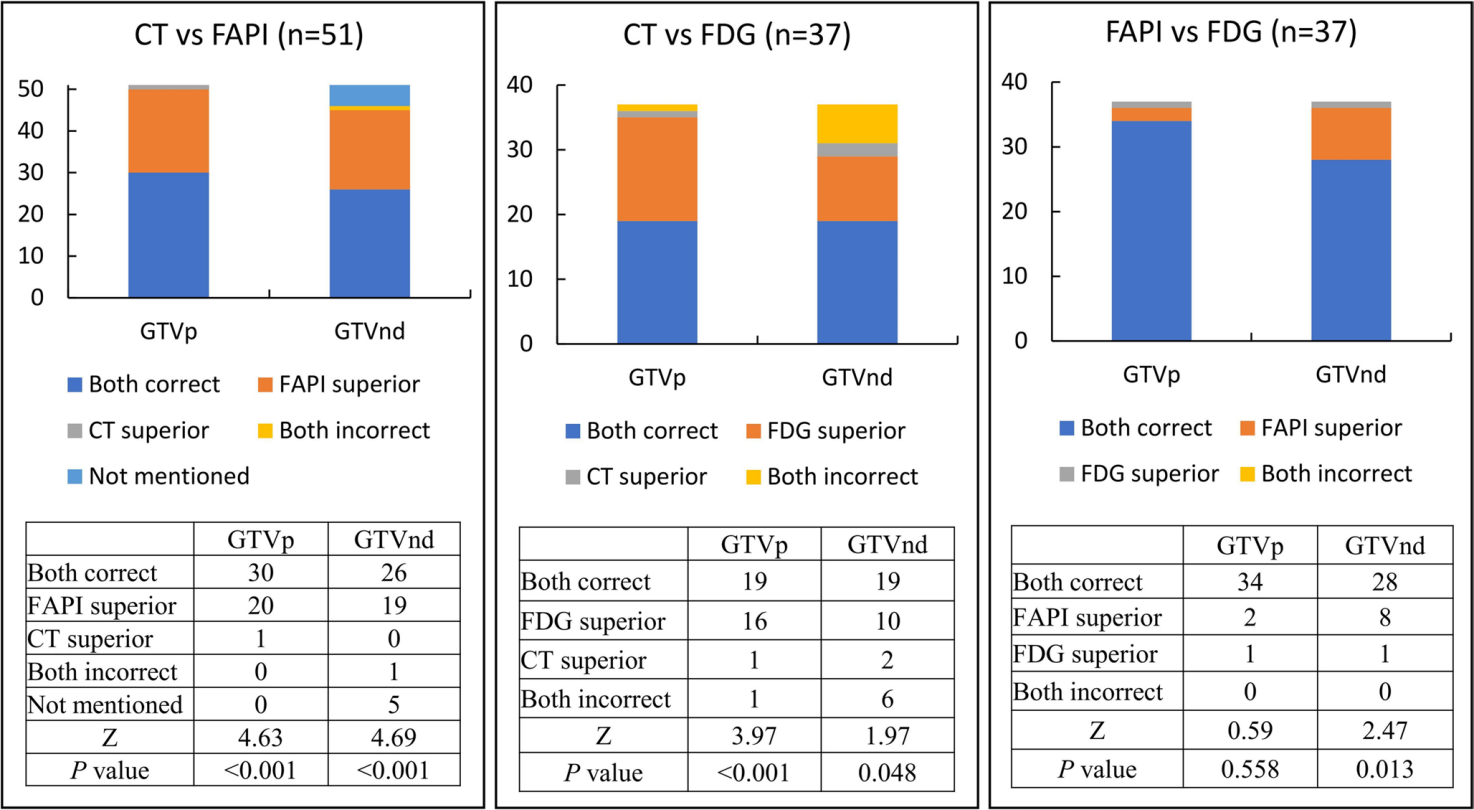
Comparison of [^18^F]AlF-NOTA-FAPI PET/CT, CE-CT and [^18^F]FDG PET/CT for the delineation of lung cancer GTVp and GTVnd. GTVp = Gross tumor volume of primary tumor; GTVnd = Gross tumor volume of lymph node; FAPI = Fibroblast activation protein inhibitor, FDG = fluorodeoxyglucose

**Fig. 4:**
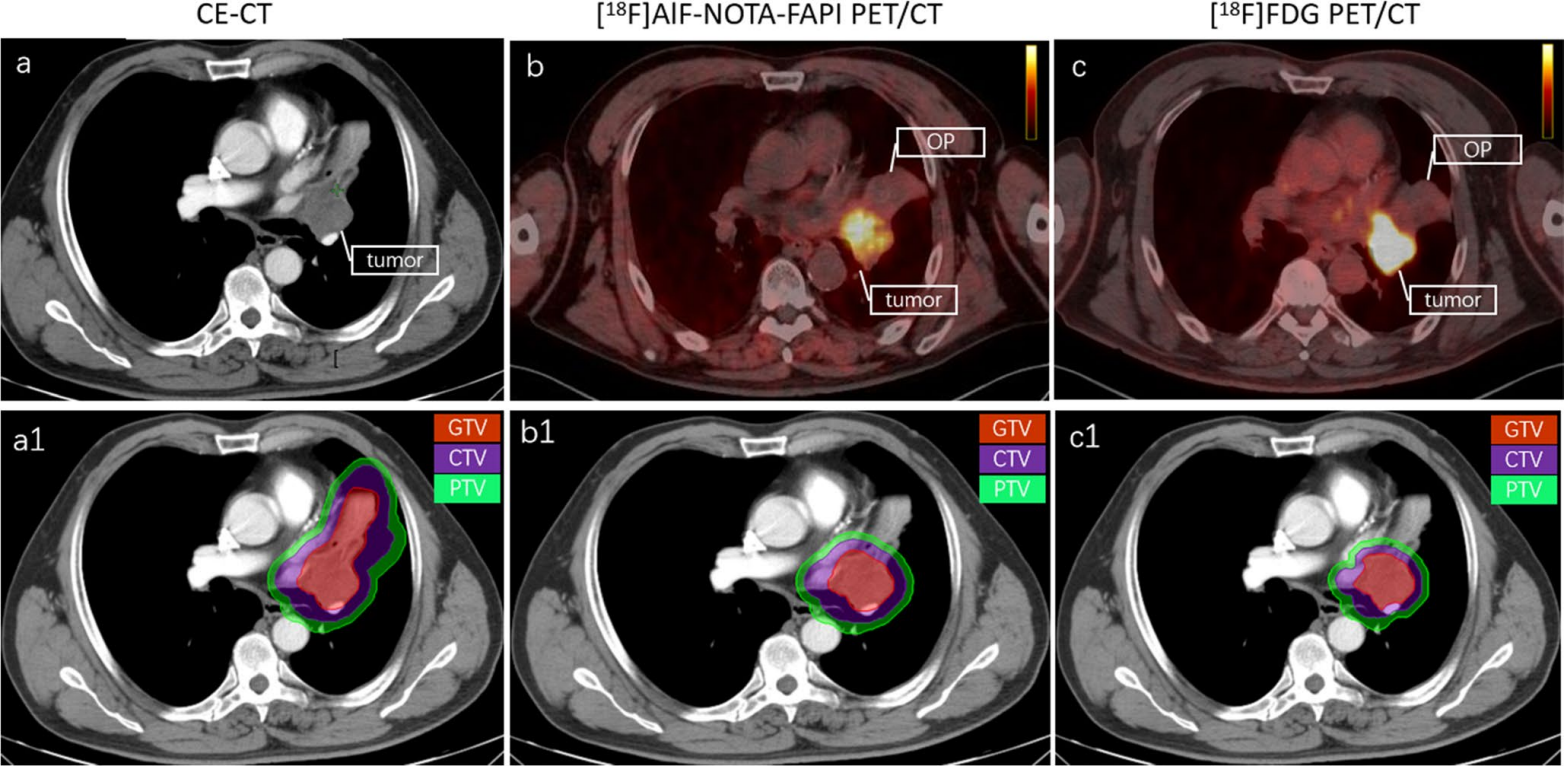
A 63-year-old man was diagnosed with central lung cancer complicated with obstructive pneumonia (OP). Both [^18^F]AlF-NOTA-FAPI and [^18^F]FDG PET/CT imaging showed clearly boundary of tumors and OP, and then more accurate and small-volume GTVp, CTVp and PTVp can be delineated. CE-CT images are difficult to distinguish the boundary between the tumor and OP, and delineated GTVp, CTVp and PTVp are larger than the true tumor volume

Figure 3; Table 2 present the GTV results for all participants based on different image-guided techniques. The accuracy rates of GTVp under the guidance of [^18^F]AlF-NOTA-FAPI PET/CT, CE-CT and [^18^F]FDG PET/CT were 98% (50/51), 61% (31/51) and 95% (35/37), respectively. The GTVp delineation under both [^18^F]AlF-NOTA-FAPI PET/CT and [^18^F]FDG PET/CT guidance was superior to that under CE-CT (both *P* < 0.001). This improvement was primarily attributed to the reduction in obstructive pneumonia and atelectasis (*n* = 18, Fig. 4). There was no statistically significant difference between the GTVp delineated under [^18^F]AlF-NOTA-FAPI PET/CT and [^18^F]FDG PET/CT guidance (*P =* 0.558).

**Table 2:** GTVp and GTVnd in the 51 study participants

NO	Primary region	GTVp				GTVnd			
		CT	FAPI PET	FDG PET	Disputed disease sites	CT	FAPI PET	FDG PET	Disputed disease sites
1	Left LC	→	→	→	/	↓	→	↓	Metastatic LN
2	Right LC	↑	→	→	OP	↑	→	→	Inflammatory LN
3	Left LC	→	→	/	/	↑	→	/	Inflammatory LN
4	Right LC	↑	→	/	OP	→	→	/	/
5	Left LC	→	↑	→	Pneumonic nodule	→	→	→	/
6	Right LC	→	→	→	/	↑	→	↑	Inflammatory LN
7	Left LC	↑	→	→	OP	↓	→	↓	Metastatic LN
8	Right LC	→	→	↓	Pulmonary metastasis	→	→	→	/
9	Left LC	→	→	→	/	→	→	→	/
10	Left LC	→	→	→	/	↑	→	↑	Inflammatory LN
11	Left LC	→	→	→	/	↓	→	→	Metastatic LN
12	Right LC	↑	→	→	OP	→	→	↑	Inflammatory LN
13	Left LC	↑	→	→	OP	→	→	→	/
14	Left LC	→	→	→	/	→	→	→	/
15	Left LC	↑	→	→	OP	↓	→	→	Metastatic LN
16	Right LC	→	→	→	/	↓	→	→	Metastatic LN
17	Left LC	→	→	→	/	→	→	→	/
18	Left LC	→	→	→	/	→	→	→	/
19	Right LC	↑	→	→	OP	→	→	→	/
20	Left LC	→	→	→	/	↓	→	→	Metastatic LN
21	Left LC	→	→	/	/	→	→	/	/
22	Left LC	↑	→	→	OP	↑	→	→	Inflammatory LN
23	Right LC	→	→	/	/	/	/	/	/
24	Left LC	→	→	→	/	→	→	↑	Inflammatory LN
25	Right LC	↑	→	→	OP	↓	→	↓	Metastatic LN
26	Left LC	→	→	/	/	→	→	/	/
27	Left LC	→	→	→	/	→	→	→	/
28	Right LC	→	→	/	/	↓	→	/	Metastatic LN
29	Right LC	↑	→	→	OP	→	→	→	/
30	Right LC	↑	→	→	OP	→	→	→	/
31	Left LC	↑	→	→	OP	↓	↓	→	Metastatic LN
32	Right LC	↑	→	/	/	↓	→	/	Metastatic LN
33	Right LC	→	→	/	/	/	/	/	/
34	Left LC	→	→	/	/	/	/	/	/
35	Right LC	→	→	/	/	/	/	/	/
36	Right LC	→	→	→	/	→	→	→	/
37	Left LC	→	→	/	/	→	→	/	/
38	Left LC	↑	→	→	OP	→	→	→	/
39	Right LC	→	→	→	/	↑	→	→	Inflammatory LN
40	Right LC	↑	→	↑	Tuberculosis	↑	→	↑	Inflammatory LN
41	Right LC	→	→	→	/	↓	→	→	Metastatic LN
42	Left LC	↑	→	→	OP	→	→	→	/
43	Left LC	↑	→	→	OP	→	→	→	/
44	Right LC	↑	→	→	OP	→	→	→	/
45	Left LC	→	→	/	/	/	/	/	/
46	Right LC	↑	→	/	OP	↓	→	/	Metastatic LN
47	Right LC	→	→	/	/	→	→	/	/
48	Left LC	→	→	→	/	→	→	→	/
49	Right LC	→	→	→	/	→	→	→	/
50	Right LC	↑	→	→	OP	↓	→	→	Metastatic LN
51	Right LC	→	→	→	/	→	→	→	/

*GTVp* Primary gross tumor target volume, *GTVnd* Lymph node gross tumor target volume, *LC* lung cancer, *FDG* fluorodeoxyglucose, *FAPI* fibroblast activation protein inhibitor, *CT* Computed Tomography, *PET* Positron Emission Tomography, *LN* lymph node, → correct target volume, ↑ up target volume, ↓ down target volume, *OP* Obstructive pneumonia

**Fig. 5:**
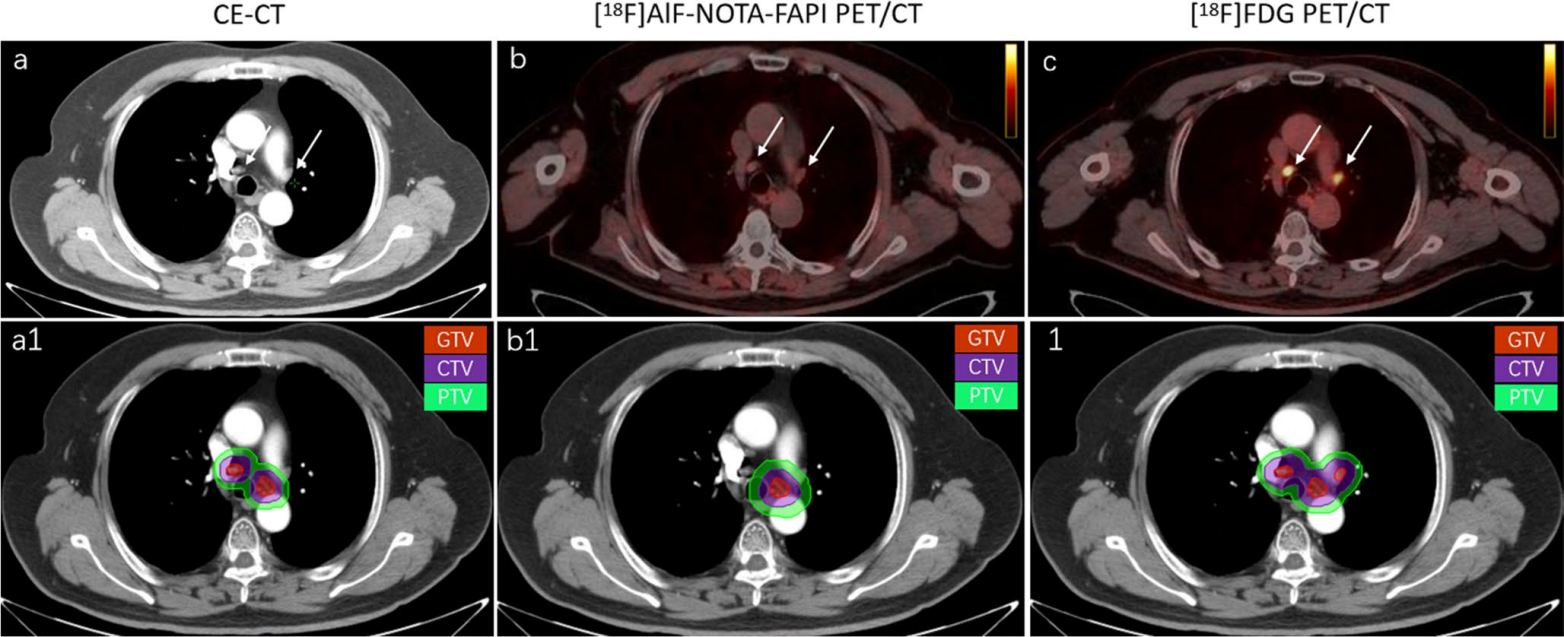
CE-CT, [^18^F]AlF-NOTA-FAPI PET/CT and [^18^F]FDG PET/CT scans of a 55-year-old man. 4R and 5 station mediastinal lymph nodes were diagnosed as true negative by CE-CT and [^18^F]FDG PET/CT, but false positive by [^18^F]FDG. Small-volume GTVnd, CTVnd and PTVnd can be delineated with the guidance of CE-CT and [^18^F]AlF-NOTA-FAPI, while larger target volume than the true lymph node was be delineated with the guidance of [^18^F]FDG PET/CT. White arrow points to lymph nodes

The accuracy rates of GTVnd under the guidance of [^18^F]AlF-NOTA-FAPI PET/CT, CE-CT and [^18^F]FDG PET/CT were 98% (45/46), 57% (26/46) and 78% (29/37), respectively. GTVnd delineated under both [^18^F]AlF-NOTA-FAPI PET/CT and [^18^F]FDG PET/CT guidance was also superior to that under CE-CT (*P* < 0.001 and *P =* 0.048, respectively). Furthermore, the GTVnd delineated under [^18^F]AlF-NOTA-FAPI PET/CT guidance was superior to that under [^18^F]FDG PET/CT guidance, *P =* 0.013 (Fig. 5).

In addition to the consistency analysis, spatial overlap between contour sets was quantified using the DSC (Online Resource 1). The mean DSC values were 0.90 ± 0.13 for GTV contours from CE-CT vs. FDG, 0.92 ± 0.12 for CE-CT and FAPI, and 0.97 ± 0.08 for FDG and FAPI. Pairwise comparisons showed that FDG vs. FAPI had significantly higher overlap than CE-CT vs. FDG (*P* = 0.019) and CE-CT vs. FAPI (*P* = 0.048), whereas no significant difference was observed between CE-CT vs. FDG and CE-CT vs. FAPI (*P* = 0.571). In addition, we included representative example images (Online Resource 2) illustrating the spatial overlap of modality-derived GTV contours to provide a visual demonstration of the DSC findings.

### Comparison of target volume based on CE-CT, [^18^F]AlF-NOTA-FAPI PET/CT, and [^18^F]FDG PET/CT

Table 3 demonstrates a comparison of target volume parameters. For GTVp, CTVp, and PTVp, the target volumes delineated based on CE-CT were significantly larger than those based on [^18^F]FDG PET/CT and [^18^F]AlF-NOTA-FAPI PET/CT due to the presence of obstructive pneumonia (*n* = 18, all *P* < 0.05). There were no statistically significant differences in GTVp, CTVp, and PTVp delineated under the guidance of [^18^F]FDG PET/CT and [^18^F]AlF-NOTA-FAPI PET/CT (all *P* < 0.05).

**Table 3:** Comparison of target volume in different images tools

	CE-CT	FDG	FAPI	*P*_all_	*P*_CT vs. FDG_	*P*_CT vs. FAPI_	*P*_FDG vs. FAPI_
GTVp	66.06 ± 10.65	56.05 ± 11.08	59.13 ± 10.26	< 0.001	0.005	0.003	0.708
GTVnd	15.33 ± 2.52	15.25 ± 2.58	15.14 ± 2.53	0.443	0.701	0.814	0.746
CTVp	155.74 ± 17.64	135.73 ± 18.00	139.61 ± 17.38	0.003	0.001	0.001	0.703
CTVnd	70.80 ± 9.33	78.11 ± 10.05	71.51 ± 9.26	0.554	0.627	0.86	0.475
PTVp	254.98 ± 24.55	224.86 ± 25.16	231.77 ± 24.03	0.003	0.001	0.002	0.461
PTVnd	136.29 ± 15.95	154.04 ± 17.11	137.49 ± 15.83	0.554	0.391	0.889	0.406
PTVall-IMRT	351.98 ± 26.87	346.95 ± 27.93	329.98 ± 26.21	0.338	0.117	0.03	0.169
PTVall-IMPT	212.74 ± 18.73	204.26 ± 19.34	196.99 ± 18.38	0.213	0.046	0.011	0.082

*GTVp* Primary gross tumor target volume, *GTVnd* Lymph node gross tumor target volume, *CTVp* Primary clinical tumor volume, *CTVnd* Lymph node clinical tumor volume, *PTVp* Primary planning tumor volume, *PTVnd* Lymph node planning tumor volume, *PTVall* The volume after the fusion of PTVp and PTVnd, *IMRT *Intensity-modulated radiation therapy, *IMPT *Intensity-modulated proton therapy, *FDG* fluorodeoxyglucose, *FAPI* fibroblast activation protein inhibitor, *CE-CT* contrast-enhanced CT

Although there were noticeable differences in the consistency of metastatic lymph node target delineation under different imaging guidance, no significant statistical differences were observed in GTVnd, CTVnd, and PTVnd delineated under CE-CT, [^18^F]FDG PET/CT, and [^18^F]AlF-NOTA-FAPI PET/CT guidance, with all *P*-values greater than 0.05.

For IMRT, PTVall-IMRT values delineated under CE-CT, [^18^F]FDG PET/CT and [^18^F]AlF-NOTA-FAPI PET/CT guidance were 351.98 ± 26.87, 346.95 ± 27.93, and 329.98 ± 26.21, respectively. The PTVall-IMRT based on CE-CT was significantly larger than that based on [^18^F]AlF-NOTA-FAPI PET/CT (*P* = 0.03), while no significant differences were observed between the other pairs.

For IMPT, PTVall-IMPT values based on CE-CT, [^18^F]FDG PET/CT and [^18^F]AlF-NOTA-FAPI PET/CT guidance were 212.74 ± 18.73, 204.26 ± 19.34, and 196.99 ± 18.38, respectively. The PTVall-IMPT based on CE-CT was significantly larger than those based on [^18^F]FDG PET/CT (*P* = 0.046) and [^18^F]AlF-NOTA-FAPI PET/CT (*P* = 0.011) respectively, while no significant differences were observed between the other pairs.

### Dose parameter comparison

In the IMRT plans, the evaluation of DVH data (Table 4) revealed that the mean doses to the heart in the CE-CT-, [^18^F]FDG PET/CT-, and [^18^F]AlF-NOTA-FAPI PET/CT-based plans were 1060.86 ± 112.74, 1107.65 ± 119.36, and 969.06 ± 100.36, respectively. The mean heart dose in the [^18^F]AlF-NOTA-FAPI PET/CT plan was significantly lower than that in the CE-CT plan (*P* = 0.02), while no significant differences were observed between the other pairs. Additionally, for the heart irradiation volumes V10, V20, and V30, the [^18^F]AlF-NOTA-FAPI PET/CT plan showed significantly lower values compared to the CE-CT plan (*P* = 0.028, 0.017 and 0.033, respectively). In the [^18^F]FDG PET/CT plan, the mean dose to the contralateral lung, as well as V5, V10, and V15, were significantly higher than those in the [^18^F]AlF-NOTA-FAPI PET/CT plan (*P* = 0.042, 0.040, 0.023 and 0.049, respectively). Similarly, in the [^18^F]FDG PET/CT plan, the V5, V10, and V15 for both lungs were significantly higher than those in the [^18^F]AlF-NOTA-FAPI PET/CT plan (*P* = 0.048, 0.027 and 0.032, respectively). No statistically significant differences were observed in the remaining DVH parameters among the radiotherapy plans developed under different imaging guidance (all *P*>0.05).

**Table 4:** Dose volume histogram parameters evaluated based on IMRT in different delineated targets

	CE-CT	FDG	FAPI	*P* value		
				CE-CT vs. FDG	CE-CT vs. FAPI	FDG vs. FAPI
D2	6555.12 ± 7.12	6561.11 ± 5.61	6549.94 ± 7.20	0.696	0.182	0.402
D98	5978.25 ± 12.41	5984.86 ± 12.82	5995.53 ± 11.35	0.604	0.041	0.144
HI	0.10 ± 0.002	0.10 ± 0.002	0.09 ± 0.002	0.610	0.046	0.167
CI	0.84 ± 0.01	0.85 ± 0.005	0.84 ± 0.007	0.993	0.443	0.150
GI	4.33 ± 0.18	4.61 ± 0.18	4.62 ± 0.17	0.000	0.000	0.116
Dmax of Spinal Cord	3662.66 ± 134.29	3811.36 ± 129.61	3738.30 ± 119.60	0.338	0.380	0.590
Heart						
Dmean	1060.86 ± 112.74	1107.65 ± 119.36	969.06 ± 100.36	0.115	0.02	0.070
V5	44.70 ± 4.74	47.85 ± 5.47	42.35 ± 4.54	0.292	0.086	0.151
V10	35.35 ± 4.07	37.40 ± 4.59	32.26 ± 3.64	0.142	0.028	0.094
V20	19.75 ± 2.63	19.71 ± 2.62	16.71 ± 2.15	0.058	0.017	0.052
V30	10.71 ± 1.43	10.94 ± 1.48	9.41 ± 1.28	0.224	0.033	0.087
Involved lung						
Dmean	1690.36 ± 87.77	1788.54 ± 87.42	1684.48 ± 88.26	0.578	0.836	0.349
V5	53.03 ± 2.39	57.16 ± 2.52	52.94 ± 2.33	0.284	0.929	0.126
V10	43.94 ± 2.13	47.59 ± 2.25	43.92 ± 2.11	0.261	0.977	0.119
V15	37.29 ± 1.91	40.30 ± 1.96	37.27 ± 1.89	0.247	0.975	0.097
V20	31.53 ± 1.70	33.86 ± 1.71	31.60 ± 1.69	0.308	0.900	0.221
V25	26.63 ± 1.51	28.57 ± 1.49	26.89 ± 1.53	0.260	0.562	0.436
V30	22.89 ± 1.39	24.53 ± 1.35	23.22 ± 1.44	0.237	0.442	0.595
V35	19.91 ± 1.32	21.01 ± 1.32	20.07 ± 1.39	0.540	0.721	0.788
V40	17.20 ± 1.27	17.65 ± 1.32	17.08 ± 1.35	0.763	0.784	0.849
V45	14.54 ± 1.17	14.53 ± 1.24	14.16 ± 1.24	0.245	0.305	0.670
V50	12.09 ± 1.03	11.89 ± 1.10	11.63 ± 1.06	0.071	0.104	0.641
Uninvolved lung						
Dmean	508.85 ± 43.49	601.78 ± 54.70	492.54 ± 42.76	0.251	0.351	0.042
V5	27.27 ± 2.23	32.16 ± 2.78	26.44 ± 2.18	0.455	0.415	0.040
V10	16.62 ± 1.62	20.26 ± 1.94	15.98 ± 1.64	0.161	0.390	0.023
V15	9.22 ± 1.15	11.28 ± 1.44	8.77 ± 1.15	0.198	0.385	0.049
V20	5.91 ± 0.86	7.34 ± 1.13	5.63 ± 0.87	0.256	0.471	0.067
V25	4.14 ± 0.67	5.35 ± 0.95	4.00 ± 0.71	0.254	0.689	0.068
V30	2.85 ± 0.54	3.82 ± 0.79	2.74 ± 0.57	0.295	0.666	0.070
V35	1.81 ± 0.43	2.48 ± 0.61	1.63 ± 0.42	0.388	0.358	0.066
V40	1.04 ± 0.31	1.42 ± 0.42	0.90 ± 0.29	0.596	0.263	0.100
V45	0.56 ± 0.21	0.76 ± 0.27	0.46 ± 0.18	0.691	0.198	0.114
V50	0.28 ± 0.13	0.40 ± 0.16	0.21 ± 0.11	0.668	0.151	0.150
Lungs						
Dmean	1066.59 ± 59.18	1160.95 ± 65.16	1054.96 ± 58.62	0.350	0.545	0.071
V5	39.46 ± 2.07	43.95 ± 2.41	38.97 ± 2.01	0.328	0.572	0.048
V10	29.57 ± 1.68	33.16 ± 1.85	29.18 ± 1.64	0.165	0.526	0.027
V15	22.48 ± 1.35	24.93 ± 1.50	22.18 ± 1.33	0.153	0.529	0.032
V20	17.98 ± 1.14	19.80 ± 1.28	17.84 ± 1.13	0.207	0.707	0.061
V25	14.72 ± 0.97	16.26 ± 1.11	14.76 ± 0.10	0.183	0.898	0.085
V30	12.28 ± 0.85	13.56 ± 0.97	12.37 ± 0.88	0.187	0.744	0.099
V35	10.33 ± 0.75	11.21 ± 0.86	10.32 ± 0.79	0.369	0.972	0.136
V40	8.65 ± 0.69	9.07 ± 0.77	8.53 ± 0.72	0.944	0.618	0.385
V45	7.15 ± 0.61	7.26 ± 0.67	6.93 ± 0.64	0.426	0.235	0.717
V50	5.85 ± 0.52	5.82 ± 0.57	5.62 ± 0.53	0.147	0.079	0.920

*IMRT* Intensity-modulated radiation therapy, dose volume histogram, *D2* the dose corresponding to 2% of the target area volume, *D98* the dose corresponding to 98% of the target area volume, *CI* target conformity index, *HI* homogeneity index, *GI* gradient index, *Vx* the volume of normal organs within which the dose is greater than xGy, *Dmax* maximum dose, *Dmean* mean dose

In the IMPT plans, the DVH data (Online Resource 3) indicated that statistically significant differences were only found in the V45 and V50 of the ipsilateral lung between the CE-CT and [^18^F]FDG PET/CT plans (*P* = 0.044 and 0.037, respectively). No statistically significant differences were observed in the remaining DVH parameters among the radiotherapy plans developed under different imaging guidance (all *P*>0.05).

## Discussion

In this study, we found that [^18^F]AlF-NOTA-FAPI PET/CT, [^18^F]FDG PET/CT, and CE-CT provide complementary information, with [^18^F]AlF-NOTA-FAPI PET/CT demonstrating the highest accuracy for radiotherapy target delineation, followed by [^18^F]FDG PET/CT and then CE-CT. The FAP-specific PET tracer [^18^F]AlF-NOTA-FAPI offers an effective theragnostic approach for radiotherapy planning in lung cancer, enabling high target accuracy while reducing radiation exposure to critical organs, thereby supporting the delivery of precise and potentially less low-toxic radiotherapy.

Importantly, [^18^F]FDG PET/CT may show low or absent uptake in certain lung cancer subtypes, such as lepidic adenocarcinoma (formerly bronchioloalveolar carcinoma), typical and atypical carcinoid tumors, and mucinous adenocarcinoma, leading to underestimation or even omission of tumor extent despite the presence of viable disease [24–26]. As FAP expression in cancer-associated fibroblasts is often preserved or increased in these entities [27], FAPI PET/CT may provide higher lesion conspicuity and a more reliable depiction of true tumor burden [6], and could therefore be particularly useful for target delineation in patients with these histologies. Although our cohort was not specifically enriched for these rare subtypes and was not powered for dedicated subgroup analyses, this represents an important clinical scenario in which [^18^F]AlF-NOTA-FAPI PET/CT-guided planning may offer additional advantages over [^18^F]FDG PET/CT and warrants further investigation.

Unlike previous studies, our research did not delineate target volumes directly on PET/CT fusion images [19]. Instead, we used the imaging results of CE-CT, [^18^F]AlF-NOTA-FAPI, and [^18^F]FDG PET/CT to outline the target volumes on large-bore simulation CT scans for primary tumors and metastatic lesions. In routine clinical practice, radiotherapy planning is primarily based on simulation CT images. Considering that PET/CT images are subjected to partial volume effects and background uptake in normal tissues, determining the actual tumor extent requires exploration of different SUV thresholds. The parameter-setting process is complex, operator-dependent and variable among individuals, increasing the difficulty and unpredictability of PET-based target delineation. Therefore, in the present study we used the different imaging modalities to localise tumors and metastatic sites, but still delineates the radiotherapy target volumes on contrast-enhanced simulation CT scans.

This study revealed significant differences in the GTVp when delineated using CE-CT, [^18^F]AlF-NOTA-FAPI PET/CT, and [^18^F]FDG PET/CT. Missed diagnoses and misdiagnoses of lesions are critical issues affecting the accuracy of target delineation. Technological advancements have improved the precision of radiotherapy targeting and reduced unintended irradiation of surrounding normal tissues. The improved outcomes in lung cancer are likely attributable in part to newer diagnostic and radiotherapy techniques, such as PET-guided radiotherapy target volume delineation and the use of IMRT or IMPT. Lung cancer, particularly central-type lung cancer, is often accompanied by obstructive pneumonia [28]. This study found that obstructive pneumonia is a primary cause of inaccuracies in GTVp and PTV delineation when guided by CE-CT. Based on the findings of this study, it is recommended that for lung cancer patients with obstructive pneumonia, either [^18^F]FDG PET/CT or [^18^F]AlF-NOTA-FAPI PET/CT should be additionally performed to improve target delineation accuracy.

Involved-field radiotherapy for lung cancer is currently the standard for target delineation recommended by the RTOG guidelines. The most challenging aspect to standardize is the delineation of the GTVnd. Missed diagnosis of metastatic lymph nodes is one of the primary reasons for recurrence and metastasis in lung cancer [29, 30]. Therefore, accurate diagnosis of metastatic lymph nodes is crucial. Current studies report that the diagnostic accuracy rates of [^18^F]FDG PET/CT and [^18^F]AlF-NOTA-FAPI PET/CT for metastatic lymph nodes are 54%−83% 79%−96% [6, 31]. A meta-analysis reported that the sensitivity and specificity of CE-CT for detecting mediastinal lymph node metastases ranged from 20% to 81% and 44%−100%, respectively [32]. However, there is no reported research on [^18^F]AlF-NOTA-FAPI PET/CT-guided GTVnd delineation for metastatic lymph nodes in lung cancer. Although no statistically significant differences were found in target volumes, there were notable discrepancies in the consistency of GTVnd delineation under different imaging modalities. Analysis of the data revealed that CE-CT was more likely to miss metastatic lymph nodes, while [^18^F]FDG PET/CT tended to over-delineate nodal drainage regions. In contrast, GTVnd delineation based on [^18^F]AlF-NOTA-FAPI PET/CT was the most accurate. As reported in the literature, the diagnostic accuracy of [^18^F]AlF-NOTA-FAPI PET/CT for mediastinal lymph nodes is significantly higher than that of [^18^F]FDG PET/CT [6]. Therefore, based on the findings of this study, it is recommended that patients with suspected lymph node metastasis undergo both [^18^F]AlF-NOTA-FAPI and [^18^F]FDG PET/CT, with [^18^F]AlF-NOTA-FAPI PET/CT being the minimum requirement.

For target volumes, although there were significant discrepancies in the consistency of metastatic lymph node delineation between modalities, no statistically significant differences were observed in the GTVnd, CTVnd and PTVnd between CE-CT-, [^18^F]FDG PET/CT-, and [^18^F]AlF-NOTA-FAPI PET/CT-based plans. This likely reflects the fact that, within the same patient cohort, some nodal target volumes were overestimated while others were underestimated, resulting in non-significant differences after statistical analysis. In contrast, due to the high incidence of obstructive pneumonia in lung cancer patients, significant differences were observed in the GTVp, CTVp and PTVp. Additionally, the differences in the PTVall in this study were primarily attributed to obstructive pneumonia.

By analyzing the DVH, the results revealed that in IMRT plans, the mean doses to the heart, bilateral lungs, and contralateral lung were lower when the target volumes were delineated under the guidance of [^18^F]AlF-NOTA-FAPI PET/CT. In contrast, the differences were less pronounced in IMPT plans, primarily because the prescription dose in IMPT plans was delivered to the CTV, whereas in IMRT plans, it was delivered to the PTV. However, in clinical practice, IMRT remains more widely used than IMPT due to limitations in cost and availability. Therefore, [^18^F]AlF-NOTA-FAPI PET/CT–guided IMRT plans that reduce doses to normal organs may translate into lower radiation-induced toxicity and provide useful guidance for the selection and optimization of image-guided radiotherapy techniques.

Although the differences in PTV volumes between imaging modalities were statistically significant, small absolute changes are unlikely to be clinically meaningful in isolation. Their potential importance lies in the abservation that, in our cohort, smaller FAPI-guided PTVs were sonsistently accompanied by reductions in lung and heart doses, which may help dosimetrists to better satisfy standard dose constraints. As this is a planning study without clinical outcome data, these findings should be regarded as exploratory and hypothesis-generating.

Beyond geometric and dosimetric considerations, our findings also have implications from an immunologic perspective. Radiation-induced lymphopenia has increasingly been recognized as an important determinant of treatment outcome in lung cancer, with several studies showing that larger irradiation volumes and higher integral or low-dose exposure are associated with more pronounced post-treatment lymphocyte depletion and worse survival after radical thoracic radiotherapy [33, 34]. Cho et al. demonstrated that severe treatment-related lymphopenia was associated with reduced efficacy and shorter survival in patients receiving immune checkpoint inhibitors for advanced NSCLC [35], underscoring the importance of preserving host immune competence in the era of chemo- and immunoradiotherapy. In this context, the FAPI-guided plans in our study lowered lung low-dose parameters and cardiac doses compared with CE-CT- and/or FDG-based plans. By improving the accuracy of target delineation and thereby limiting unnecessary irradiation of normal lung and thoracic tissues involved in immune cell trafficking, [^18^F]AlF-NOTA-FAPI PET/CT-based planning may help to mitigate radiation-induced lymphopenia and better preserve systemic immune function in patients receiving combined chemo- or immuno-radiotherapy, thereby complementing conformal techniques such as IMRT, proton therapy and SBRT to improve the overall therapeutic ratio of thoracic radiotherapy.

Although SBRT has become the standard of care for many patients with early-stage, node-negative NSCLC with tumors ≤ 5–6 cm [36], conventionally fractionated thoracic radiotherapy remains widely used in routine practice, particularly in patients with central tumors, nodal involvement, SCLC or more advanced disease [37–39], as represented in our cohort. The present findings therefore primarily inform the optimization of conventionally fractionated radiotherapy. Nevertheless, the improved conspicuity of the primary tumor and the clearer separation of tumor from obstructive pneumonia or inflammatory changes provided by [^18^F]AlF-NOTA-FAPI PET/CT may also be particularly valuable in the SBRT setting, where margins are tight and dose gradients are steep. More accurate, biologically informed target delineation with FAPI PET/CT could help to reduce geographic miss and further spare surrounding normal lung in early-stage patients treated with SBRT, a hypothesis that warrants confirmation in dedicated SBRT-focused prospective studies.

This study still has some limitations. Firstly, there is a lack of pathological gold standards for assessing the accuracy of target delineation. Second, the overall sample size was modest (*n* = 51), and only 37 patients underwent both [^18^F]AlF-NOTA-FAPI and [^18^F]FDG PET/CT. As a result, the statistical power is limited, particularly for comparisons involving [^18^F]FDG PET/CT, and the study is not powered to detect small differences or to support detailed subgroup analyses; our findings should therefore be regarded as hypothesis-generating and require confirmation in larger cohorts. Third, this work was conceived as a planning and dosimetric comparison rather than an outcome study; systemic therapy regimens (e.g. concurrent chemoradiotherapy, sequential chemotherapy and consolidative immunotherapy) were not prospectively collected or stratified, and the potential interaction between [^18^F]AlF-NOTA-FAPI PET/CT-guided planning and specific systemic treatment strategies therefore remains to be addressed in dedicated prospective clinical trials. In addition, we did not perform a formal inter-observer reproducibility analysis of target delineation; however, all contours were reviewed in consensus by two experienced radiation oncologists, which may partially mitigate, but does not eliminate, potential observer bias. Furthermore, the data reported in this study are from a single center, lacking multi-center comparative study data. Finally, some literature has reported that [^18^F]FDG PET/CT-guided adaptive radiotherapy in the later stages can significantly improve local control rates and prolong survival time. Therefore, prospective clinical trials are necessary to further validate the application value of [^18^F]AlF-NOTA-FAPI PET/CT-guided adaptive radiotherapy in the later stages.

## Conclusion

Comparative studies have revealed that [^18^F]AlF-NOTA-FAPI PET/CT holds significant potential for clinical application in target delineation for lung cancer, particularly in accurately defining lung cancer with obstructive pneumonia and metastatic lymph nodes. Additionally, it offers better protection for normal organs in IMRT. We believe that [^18^F]AlF-NOTA-FAPI PET/CT will demonstrate broad clinical utility in radiation oncology and warrants further investigation into its theragnostic role in future clinical applications.

## Supplementary Information

Below is the link to the electronic supplementary material.

Supplementary Material 1 (PDF 99.3 KB)

Supplementary Material 2 (PDF 131 KB)

Supplementary Material 3 (PDF 112 KB)

## Data Availability

The datasets generated during and/or analyzed during the current study are available from the corresponding author on reasonable request.
