# Supplementary material for: Value of [18F]AlF-NOTA-FAPI PET/CT in guiding radiotherapy planning for stage I-IIIC lung cancer: A comparison with contrast-enhanced CT and [18F]FDG PET/CT
Source: Eur J Nucl Med Mol Imaging. 2026 Feb 5;53(6):3760–74. doi: 10.1007/s00259-025-07753-7 (PMC13121188; doi:10.1007/s00259-025-07753-7)
Supplement: Supplementary file 1 — Supplementary Material 1 (PDF 99.3 KB) [file 259_2025_7753_MOESM1_ESM.pdf]

Article title

Value of $[^{18}F]$AlF-NOTA-FAPI PET/CT in Guiding Radiotherapy Planning for Stage I–IIIC Lung Cancer: A Comparison with Contrast-enhanced CT and $[^{18}F]$FDG PET/CT

Journal

European Journal of Nuclear Medicine and Molecular Imaging (EJNMMI)

Authors

Jingjie Qin; Chengqiang Li; Yong Huang; Yuqin Jin; Xiaoshan Liu; Xudong Hu; Jian Zhu; Junya San; Hongbo Wu; Xue Meng; Jinming Yu; Yuchun Wei.

Corresponding author

Yuchun Wei, MD, PhD — Department of Radiation Oncology, Shandong Cancer Hospital and Institute, Shandong First Medical University and Shandong Academy of Medical Sciences, No. 440 Jiyan Road, Jinan, Shandong 250117, China. Email: ycwei@email.sdfmu.edu.cn

Online Resource 1 DSC and pairwise comparisons

Contour-pair category	DSC(mean±SD)	Comparison	*P* value
CE-CT-FDG	0.90 ± 0.13	vs CE-CT-FAPI	0.571
CE-CT-FAPI	0.92 ± 0.12	vs FDG-FAPI	0.048
FDG-FAPI	0.97 ± 0.08	vs CE-CT-FDG	0.019

Note: DSC: dice similarity coefficient; SD: standard deviation; FDG, fluorodeoxyglucose; FAPI, fibroblast activation protein inhibitor; CE-CT,contrast-enhanced CT; CE-CT-FDG, CE-CT-FAPI, and FDG-FAPI denote DSC calculated between GTV contour sets delineated on CE-CT and [^{18}F]FDG PET/CT, CE-CT and [^{18}F]AlF-NOTA-FAPI PET/CT, and [^{18}F]FDG PET/CT and [^{18}F]AlF-NOTA-FAPI PET/CT, respectively.
