# Supplementary material for: Value of [18F]AlF-NOTA-FAPI PET/CT in guiding radiotherapy planning for stage I-IIIC lung cancer: A comparison with contrast-enhanced CT and [18F]FDG PET/CT
Source: Eur J Nucl Med Mol Imaging. 2026 Feb 5;53(6):3760–74. doi: 10.1007/s00259-025-07753-7 (PMC13121188; doi:10.1007/s00259-025-07753-7)
Supplement: Supplementary file 2 — Supplementary Material 2 (PDF 131 KB) [file 259_2025_7753_MOESM2_ESM.pdf]

Article title

Value of [^{18}F]AlF-NOTA-FAPI PET/CT in Guiding Radiotherapy Planning for Stage I–IIIC Lung Cancer: A Comparison with Contrast-enhanced CT and [^{18}F]FDG PET/CT

Journal

European Journal of Nuclear Medicine and Molecular Imaging (EJNMMI)

Authors

Jingjie Qin; Chengqiang Li; Yong Huang; Yuqin Jin; Xiaoshan Liu; Xudong Hu; Jian Zhu; Junya San; Hongbo Wu; Xue Meng; Jinming Yu; Yuchun Wei.

Corresponding author

Yuchun Wei, MD, PhD — Department of Radiation Oncology, Shandong Cancer Hospital and Institute, Shandong First Medical University and Shandong Academy of Medical Sciences, No. 440 Jiyan Road, Jinan, Shandong 250117, China. Email: ycwei@email.sdfmu.edu.cn

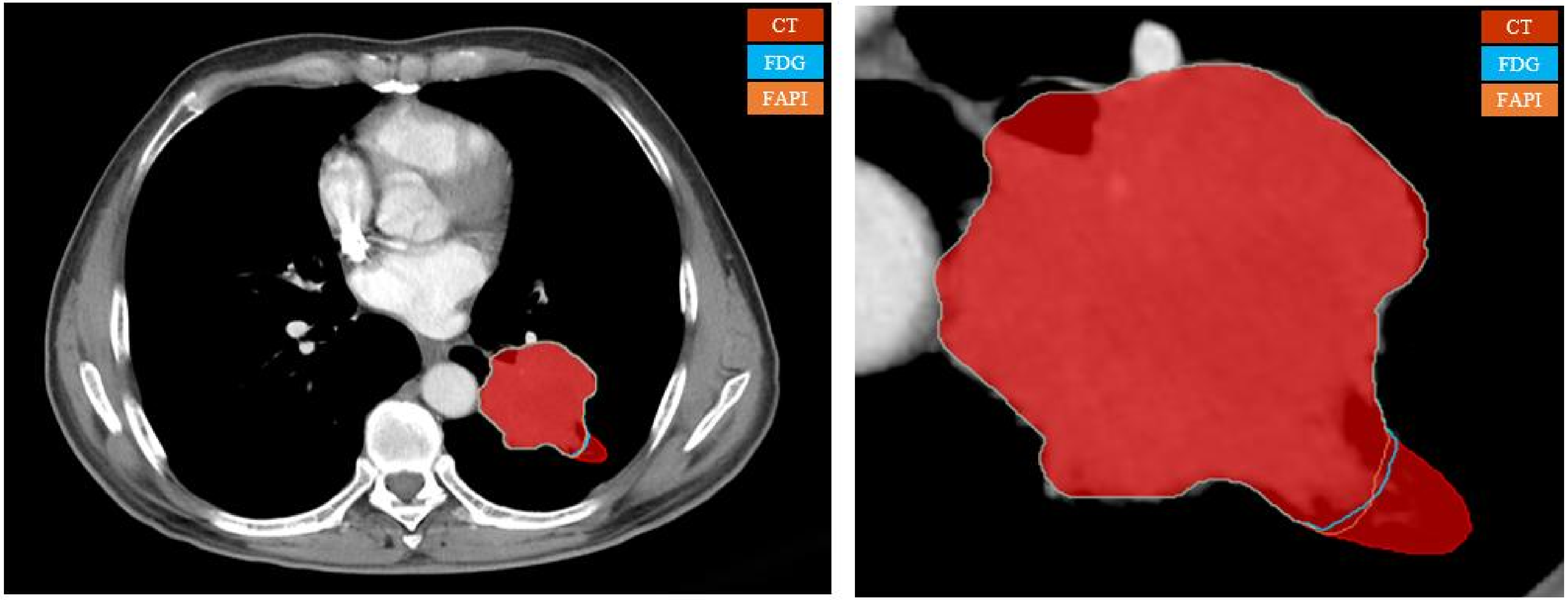

Online Resource 2. Representative example of spatial overlap among modality-derived GTV contours.Axial CE-CT images show gross tumor volume (GTV) contours delineated on CE-CT (red), [^{18}F]FDG PET/CT (blue), and [^{18}F]AlF-NOTA-FAPI PET/CT (orange) for the same patient. The right panel provides a magnified view highlighting differences in contour extent at the tumor margin.
