# Supplementary material for: Value of [18F]AlF-NOTA-FAPI PET/CT in guiding radiotherapy planning for stage I-IIIC lung cancer: A comparison with contrast-enhanced CT and [18F]FDG PET/CT
Source: Eur J Nucl Med Mol Imaging. 2026 Feb 5;53(6):3760–74. doi: 10.1007/s00259-025-07753-7 (PMC13121188; doi:10.1007/s00259-025-07753-7)
Supplement: Supplementary file 3 — Supplementary Material 3 (PDF 112 KB) [file 259_2025_7753_MOESM3_ESM.pdf]

**Article title**

Value of [$^{18}$F]AlF-NOTA-FAPI PET/CT in Guiding Radiotherapy Planning for Stage I–IIIC Lung Cancer: A Comparison with Contrast-enhanced CT and [$^{18}$F]FDG PET/CT

**Journal**

European Journal of Nuclear Medicine and Molecular Imaging (EJNMMI)

**Authors**

Jingjie Qin; Chengqiang Li; Yong Huang; Yuqin Jin; Xiaoshan Liu; Xudong Hu; Jian Zhu; Junya San; Hongbo Wu; Xue Meng; Jinming Yu; Yuchun Wei.

**Corresponding author**

Yuchun Wei, MD, PhD — Department of Radiation Oncology, Shandong Cancer Hospital and Institute, Shandong First Medical University and Shandong Academy of Medical Sciences, No. 440 Jiyan Road, Jinan, Shandong 250117, China. Email: ycwei@email.sdfmu.edu.cn

**Online Resource 3 Dose volume histogram parameters evaluated based on IMPT in different delineated targets**

| | CT | FDG | FAPI | *P* value | | |
|---|---|---|---|---|---|---|
| | | | | CT vs FDG | CT vs FAPI | FDG vs FAPI |
| D2 | 6583.27±4.59 | 6587.11±6.01 | 6589.25±5.03 | 0.481 | 0.150 | 0.050 |
| D98 | 6059.96±9.34 | 6054.49±10.69 | 6064.47±9.58 | 0.743 | 0.456 | 0.773 |
| HI | 0.09±0.002 | 0.09±0.002 | 0.09±0.002 | 0.895 | 0.856 | 0.646 |
| CI | 0.62±0.01 | 0.60±0.01 | 0.60±0.12 | 0.038 | 0.025 | 0.683 |
| GI | 3.18±0.11 | 3.19±0.11 | 3.19±0.11 | 0.985 | 0.778 | 0.537 |
| Dmax of Spinal Cord | 3013.85±179.64 | 3216.54±190.25 | 2972.71±171.02 | 0.634 | 0.536 | 0.073 |
| Heart | | | | | | |
| Dmean | 355.50±45.46 | 376.18±48.69 | 332.14±40.31 | 0.756 | 0.197 | 0.229 |
| V5 | 13.77±1.54 | 14.63±1.67 | 13.06±1.36 | 0.693 | 0.315 | 0.425 |
| V10 | 10.07±1.21 | 10.70±1.32 | 9.55±1.07 | 0.771 | 0.352 | 0.455 |
| V20 | 6.35±0.86 | 6.73±0.95 | 5.87±0.76 | 0.806 | 0.163 | 0.195 |
| V30 | 4.21±0.65 | 4.46±0.69 | 3.85±0.57 | 0.866 | 0.135 | 0.138 |
| Involved lung | | | | | | |
| Dmean | 1414.61±84.69 | 1486.67±86.37 | 1390.42±87.28 | 0.521 | 0.327 | 0.470 |
| V5 | 41.78±2.01 | 44.98±1.95 | 41.49±2.05 | 0.681 | 0.715 | 0.267 |
| V10 | 35.81±1.89 | 38.77±1.91 | 35.64±1.97 | 0.698 | 0.806 | 0.204 |
| V15 | 30.94±1.76 | 33.57±1.81 | 30.86±1.85 | 0.634 | 0.900 | 0.186 |
| V20 | 26.80±1.65 | 28.87±1.71 | 26.67±1.71 | 0.798 | 0.788 | 0.227 |
| V25 | 23.50±1.53 | 24.74±1.59 | 23.13±1.59 | 0.524 | 0.354 | 0.512 |
| V30 | 20.75±1.42 | 21.31±1.51 | 20.15±1.47 | 0.105 | 0.093 | 0.809 |
| V35 | 18.25±1.32 | 18.51±1.43 | 17.61±1.36 | 0.062 | 0.060 | 0.952 |
| V40 | 15.98±1.22 | 16.09±1.33 | 15.41±1.26 | 0.054 | 0.069 | 0.886 |

| | | | | | | |
|---|---|---|---|---|---|---|
| V45 | 13.91±1.12 | 13.92±1.23 | 13.37±1.15 | 0.044 | 0.063 | 0.906 |
| V50 | 11.91±1.00 | 11.84±1.11 | 11.42±1.03 | 0.037 | 0.064 | 0.895 |
| Uninvolved lung | | | | | | |
| Dmean | 94.43±22.08 | 128.78±30.06 | 86.63±20.44 | 0.388 | 0.437 | 0.123 |
| V5 | 4.66±0.93 | 6.42±1.36 | 4.48±0.95 | 0.219 | 0.693 | 0.103 |
| V10 | 3.04±0.71 | 4.18±0.96 | 2.87±0.68 | 0.296 | 0.608 | 0.117 |
| V15 | 2.08±0.56 | 2.88±0.74 | 1.92±0.52 | 0.410 | 0.511 | 0.122 |
| V20 | 1.41±0.43 | 1.97±0.55 | 1.24±0.37 | 0.514 | 0.402 | 0.132 |
| V25 | 0.95±0.32 | 1.35±0.41 | 0.82±0.27 | 0.536 | 0.411 | 0.165 |
| V30 | 0.64±0.24 | 0.92±0.31 | 0.54±0.20 | 0.577 | 0.377 | 0.196 |
| V35 | 0.47±0.19 | 0.66±0.25 | 0.37±0.15 | 0.647 | 0.261 | 0.200 |
| V40 | 0.36±0.15 | 0.50±0.20 | 0.28±0.13 | 0.675 | 0.217 | 0.198 |
| V45 | 0.28±0.13 | 0.39±0.16 | 0.22±0.10 | 0.693 | 0.199 | 0.202 |
| V50 | 0.22±0.10 | 0.30±0.13 | 0.17±0.08 | 0.733 | 0.178 | 0.214 |
| Lungs | | | | | | |
| Dmean | 715.98±46.24 | 769.11±50.96 | 701.53±47.53 | 0.954 | 0.297 | 0.189 |
| V5 | 22.12±1.23 | 24.56±1.39 | 21.88±1.25 | 0.287 | 0.625 | 0.083 |
| V10 | 18.44±1.10 | 20.43±1.23 | 18.26±1.13 | 0.359 | 0.661 | 0.083 |
| V15 | 15.65±0.99 | 17.31±1.11 | 15.52±1.02 | 0.402 | 0.701 | 0.083 |
| V20 | 13.35±0.90 | 14.64±0.99 | 13.21±0.92 | 0.566 | 0.597 | 0.112 |
| V25 | 11.56±0.81 | 12.38±0.89 | 11.34±0.83 | 0.936 | 0.318 | 0.248 |
| V30 | 10.10±0.74 | 10.55±0.82 | 9.80±0.76 | 0.382 | 0.103 | 0.420 |
| V35 | 8.84±0.67 | 9.10±0.75 | 8.53±0.70 | 0.215 | 0.060 | 0.563 |
| V40 | 7.72±0.62 | 7.88±0.69 | 7.45±0.64 | 0.162 | 0.069 | 0.737 |
| V45 | 6.71±0.56 | 6.80±0.63 | 6.45±0.58 | 0.120 | 0.067 | 0.772 |

| | | | | | | |
|---|---|---|---|---|---|---|
| V50 | 5.73±0.50 | 5.77±0.56 | 5.51±0.52 | 0.087 | 0.069 | 0.835 |

Note-IMPT, Intensity-modulated proton therapy; dose volume histogram; D2, the dose corresponding to 2% of the target area volume; D98, the dose corresponding to 98% of the target area volume; CI, target conformity index; HI, homogeneity index; GI, gradient index; Vx=the volume of normal organs within which the dose is greater than xGy; Dmax, maximum dose; Dmean, mean dose.
